# Curcumin and Vitamin D Supplement Attenuates Knee Osteoarthritis Progression in ACLT + MMx Rat Model: Effect on Cartilage Protection and Pain Reduction

**DOI:** 10.3390/nu17020349

**Published:** 2025-01-19

**Authors:** Lokesh Kumar Mende, Yaswanth Kuthati, Chih-Shung Wong

**Affiliations:** 1 Department of Anesthesiology, Cathay General Hospital, Taipei 280, Taiwan; lokeshyv66@gmail.com (L.K.M.); yaswanthk1987@gmail.com (Y.K.); 2 National Defense Medical Center, Institute of Medical Sciences, Taipei 280, Taiwan

**Keywords:** knee osteoarthritis, Toll-like receptor, anti-inflammation, fibroblast growth factor-23, pain, inflammation, synoviocytes, metalloprotease

## Abstract

**Background:** Knee osteoarthritis (OA) is a common and debilitating disorder marked by joint degradation, inflammation, and persistent pain. This study examined the possible therapeutic effects of curcumin and vitamin D on OA progression and pain in a rat knee OA model by anterior cruciate ligament transection and meniscectomy (ACLT + MMx). **Methods:** Male Wistar rats were categorized into five groups: control, curcumin-treated (100 mg/kg/day), vitamin D-treated (25 µg/kg/day), a combination of vitamin D and curcumin, and sham-operated. All supplements were administered orally on a daily basis for 12 weeks. Pain behaviors were assessed, serum biomarkers were measured, and knee histology was examined. **Results:** Both curcumin and vitamin D independently reduced pain, while the combined group exhibited better analgesic effects. Serum inflammatory cytokines demonstrated a decrease in pro-inflammatory cytokines and an elevation in anti-inflammatory cytokine interleukin-10 (IL-10) in the supplement groups. The antioxidative markers were partially recovered by curcumin and vitamin D supplement. However, the oxidative stress marker Cartilage Oligomeric Matrix Protein (COMP) was significantly reduced. Histology analysis revealed a preservation of joint architecture and cartilage integrity and decreased synovium inflammation in the groups treated with curcumin and vitamin D. **Conclusions:** Our findings indicate a dual mechanism that encompasses the role of anti-inflammation and antioxidation on knee OA progression and pain reduction, underscoring the potential of these natural chemicals as therapeutic agents for knee OA; curcumin and vitamin D supplement may be added in delaying knee OA progression and associated pain management in clinical patient care.

## 1. Introduction

Knee osteoarthritis (OA) is characterized by joint pain, joint inflammation, joint space narrowing, synoviocyte damage, and cartilage damage [1]. Chronic and severe OA patients are finally advised to undergo joint replacement therapy for pain relief [2]. In knee OA, the articular cartilage and the subchondral bones underneath are extremely affected [3]. Type-X collagen and matrix metalloproteinase (MMP) levels are high and used as molecular markers for OA and other characteristics such as calcification, disorientation, and osteophyte formation [4]. Various pathways bind to receptors and phosphorylate SMAD3 to block the matrix metalloproteinase-13 (MMP-13) expression [5]. In a different study, reduced levels of TGF-β have been linked to increased cartilage damage in osteoarthritis models, indicating its crucial function in cartilage repair and maintenance. TGF-β knock-out studies have shown increased MMP-13 production, emphasizing its role in preventing cartilage breakdown [6,7]. The joint cavity is packed with the synovial membrane arranged by fibroblast-like cells and secrets hyaluronic and lubricin [8,9,10], which are essential for synovial fluid viscosity and the lubrication of the articular cartilage [11,12]. Reactive oxygen species (ROS) are the primary cause of cartilage damage in knee OA [13,14,15]. The imbalance between the anabolism and catabolism of chondrocytes caused by MMPs may damage the chondrocytes [16,17]. Several reports demonstrated that natural products including curcumin (from turmeric), omega-3 fatty acids (from fish oil and flaxseed), ginger, boswellia serrata (Indian frankincense), avocado/soybean unsaponifiable (ASU), resveratrol (from grapes and red wine), green tea extract (EGCG), chondroitin sulfate, glucosamine, and vitamin D overcame the inflammatory and pain-related responses in synovial tissue via the Toll-like receptor (TLR) signaling pathway to release the pro-inflammatory cytokines and ROS, and attenuated chondrocytes catabolic activity at the knee joint [18,19,20]. Tumor necrosis factor-alpha (TNF-α) and interleukin-6 (IL-6) are two inflammatory cytokines released by chondrocytes and synoviocytes and also play crucial roles in OA progression [21,22]. Wang et al. found that the cartilage damage increased the production of ROS and triggered important pathways to release pro-inflammatory cytokines and pain-related proteins, which worsen knee OA progression [23]. Vitamin D regulates cartilage integrity by promoting cartilage repair and reducing degradation, joint pain, and inflammation in OA [24,25]. Emerging evidence supports vitamin D’s regulatory function in cartilage repair, joint pain reduction, and inflammation, highlighting its capacity to influence inflammatory pathways and oxidative stress markers linked with OA progression [26]. Animal studies suggested that vitamin D supplements could inhibit OA development, joint deformation, and cartilage volume [27]. Huhtakangas et al. showed that vitamin D produced the anti-inflammation and antiproliferation of synoviocytes in RA and OA patients [28]. Recent studies also suggest that vitamin D might influence knee OA by affecting bone mineralization, inflammation, and cartilage health. However, its exact role in knee OA and pain management remains under investigation, with some studies indicating potential benefits in reducing inflammation and pain in OA-affected joints.

Curcumin is widely used in traditional medicine for its numerous pharmacological effects, including anti-inflammatory, antioxidant, and anti-fibrotic properties [29,30,31]. Curcumin, a polyphenol produced from turmeric, has also received attention for its potent anti-inflammatory and antioxidant effects, with emerging data having supported its capacity to attenuate cartilage deterioration, regulate inflammatory mediators, and alleviate symptoms in OA [32,33]. Recent research has made significant progress in using curcumin to treat knee OA by delaying disease progression and promoting cartilage repair [34]. Curcumin modulates inflammatory responses by reducing the levels of cyclo-oxygenase-2 (COX-2), lipo-oxygenase, and inducible nitric oxide synthase (iNOS), and cytokines such as TNF-α, IL-1, IL-6, and IL-10 [35]. Additionally, curcumin inhibits apoptosis by modulating various cellular pathways, including reducing interleukin-1-beta (IL-1β)-induced changes in the expression levels of Bcl-2, Bcl-XL, Bax, and caspase-3 and suppressing the phosphorylation of p38 and JNK while increasing ERK and AKT expression [36,37]. Furthermore, it reduces inflammation by blocking the MyD88/TLR4/NF-κB signaling pathway, highlighting its therapeutic potential for knee OA [38]. In addition, curcumin supplements have been shown to lower serum malondialdehyde (MDA) levels and increase antioxidant enzyme levels, further supporting its anti-osteoarthritic effects. Given these benefits, curcumin could be a viable option for managing knee OA via its antioxidative property to delay OA progression. Some reports found that curcumin might help alleviate symptoms of OA, such as pain and inflammation, and protect against cartilage degradation.

Combining vitamin D and curcumin for knee OA management may offer synergistic benefits. The significance of dietary treatments and nutrients aligned perfectly with the discussion on OA management techniques by alleviating oxidative stress and inflammation [39]. The present understanding of the roles of vitamin D and curcumin in the treatment of OA emphasizes their synergistic benefits in lowering oxidative stress and inflammation [32,33,40,41,42]. The synergistic effects of vitamin D and curcumin in lowering oxidative stress and inflammation have been highlighted, emphasizing their potential as a comprehensive approach to managing OA [32,41]. In this context, vitamin D supports bone health and potentially reduces inflammation, while curcumin provides anti-inflammatory and antioxidant effects. Together, they could effectively delay knee OA progression by reducing inflammation, protecting cartilage, and improving joint function. In vivo and in vitro studies have not yet explored the potential association of curcumin and vitamin D on knee OA progression, pain relief, and chondrocyte protection. In this study, we evaluated the efficiency of curcumin and vitamin D on knee OA progression and pain reduction. Our data show that an oral curcumin and vitamin D supplement provided anti-inflammatory and antioxidative stress, which lowered OA discomfort, cartilage loss, joint inflammation, and MMP expression.

## 2. Materials and Methods

**Figure 1:**
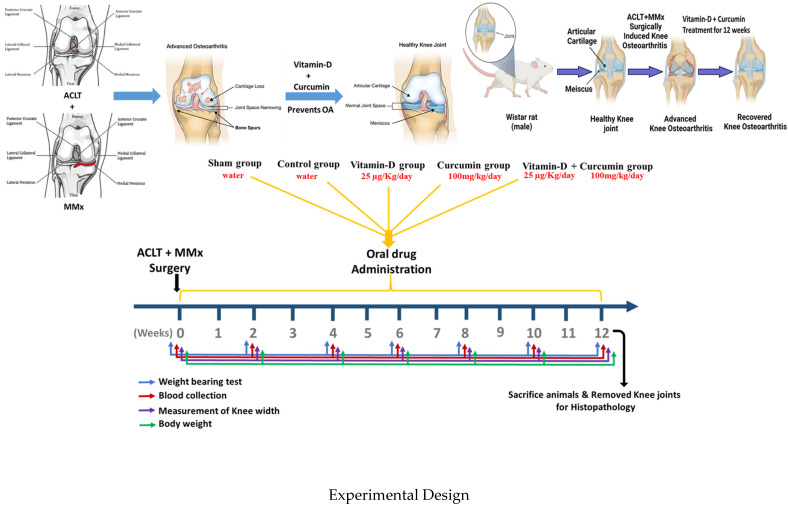
The experimental design and time frame of the study.

This study examined the effects of curcumin and vitamin D in the treatment of knee OA both in vitro and in vivo. Figure 1 describes the experimental design of our study using the ACLT + MMx to generate OA. The treatment groups included sham (water), control (water), curcumin (100 mg/kg/day) [43], vitamin D (25 µg/kg/day), and a combination of vitamin D + curcumin (25 µg/kg/day + 100 mg/kg/day). These treatments were administered orally and daily for 12 weeks. Throughout the experiment, body weight, knee width measurements, weight-bearing tests, and blood samples were collected at specific time points (weeks 0, 4, 8, and 12) [44,45,46,47]. Subsequent to a period of twelve weeks, the rats under study were sacrificed, followed by the removal of their knee joints for histopathological analysis to evaluate the effects of the treatments on joint health.

### 2.1. Animal Model: ACTL + MMx-Induced Knee OA

The procedure was performed in accordance with previous reports [48,49]. Male Wistar rats (330–350 g) were sedated with 5% isoflurane in an induction chamber. ACLT + MMx was performed on the rat’s right knee; a povidone–iodine solution was used to sterilize the part after the removal of hair. A cut was made on the inner side of the joint capsule, and the front essential ligament was cut with a scalpel. The medial meniscus (a C-shaped piece of cartilage that acts as a shock absorber) was then removed with surgical scissors. Finally, the joint capsule was sutured closed with 4-0 vicarly-monofilament nylon. To avoid infection, the wound was sterilized, and cefazolin was administered intramuscularly at a dose of 100 mg/kg/day for three days. The sham group rats received the same skin incision with no ACLT and medial meniscus removed.

### 2.2. Administration of Drugs

The experimental design was planned in accordance with the methods listed above. Vitamin D and curcumin were dissolved in water. Thirty rats were divided randomly into five groups: sham (water), control (water), vitamin D (25 µg/kg/day), curcumin (100 mg/kg/day), and vitamin D + curcumin (25 µg/kg/day + 100 mg/kg/day) (*n* = 6, each). The drugs were given orally and daily for 12 weeks.

### 2.3. Knee Width and Weight-Bearing Test Measurement

The knee width was measured using a caliper every two weeks after surgery, with the contralateral knee width (in mm) serving as the control. Weight-bearing assessments were also conducted biweekly, utilizing an in-capacitance tester to evaluate paw static weight bearing, which helped predict changes in postural equilibrium caused by knee OA. During the test, rats were positioned on their hind paws in boxes equipped with an inclined plane set at a 65° angle from the horizontal. After calibration, the machine measured the weight applied by each hind limb. The difference in force (Δ Force, in grams) between the control and the operated hind leg was recorded. This variation in weight distribution reflected the pain and discomfort associated with OA in the operated limb.

### 2.4. Histopathology Examination of the Knee Joints

All rats were euthanized after 12 weeks within their respective groups. The knee joints were harvested and fixed in 10% formalin for three days before undergoing decalcification in a 12.5% EDTA disodium solution (pH 7.0) for four weeks. The decalcified joints were then embedded in paraffin blocks, and serial histological sections, 5 μm thick, were prepared. Morphological alterations were assessed using toluidine blue and rapid green staining. The stained sections were photographed with a ZEISS Axiom picture system and ZEN lite 2.6 for the blue edition. The modified OA Research Society International (OARSI) scoring system was employed to evaluate the severity of articular cartilage destruction to the medial tibial plateau. Cartilage changes were assessed using measurements of cartilage matrix width loss, tibial cartilage degradation scores, total cartilage width loss, and the zonal depth ratio of the synovial membrane.

### 2.5. Measurement of Cytokines

Prior to sacrificing the animals under study, their tail veins were utilized to draw serum samples, which were collected by centrifuging blood at 3000× *g* for 15 min and stored at −80 °C for future investigations. The total serum levels of TNFα, IL-1β, IL-6, and IL-10 were measured using an ELISA kit (Calbiochem-Nocabiochem Co., Milan, Italy), following the manufacturer’s instructions [50].

### 2.6. Measurement of Antioxidant Parameters

The oxidative-stress-related serum biomarkers superoxide dismutase (SOD), glutathione peroxidase (GPX), reduced glutathione levels (GSH), and COMP were estimated using the previous protocols [51]. ELISA kits from Abcam Co., Ltd. (Cambridge, MA, USA) were used. The recorded measurements were performed thrice based on the manufacturer’s instructions.

### 2.7. ELISA Studies

The serum markers were determined by the radioimmunoassay assay using a Cobas E411 analyzer based on the commercial ELISA kits for TNF-α, IL-1β, IL-6, and IL-10 (San Diego, CA, USA), and MMP-3, MMP-13, and *C*-terminal cross-linked telopeptides of type II collagen (CTX-II) (Abcam Co., Ltd., Cambridge, MA, USA). As specified by the manufacturer, all measurements were analyzed in triplicate. A standard curve was plotted to detect the concentrations of each independent study sample [21,22,52].

### 2.8. Statistical Examination

The data were expressed as mean ± S.D. All figures were created using GraphPad Prism version 9. Statistical analysis was performed using one-way analysis of variance (ANOVA) followed by a *t*-test for multiple group comparisons, with a *p*-value of less than 0.05 considered statistically significant. Significant differences between groups were denoted as * *p* < 0.05, ** *p* < 0.01, and *** *p* < 0.001.

## 3. Results

**Figure 2:**
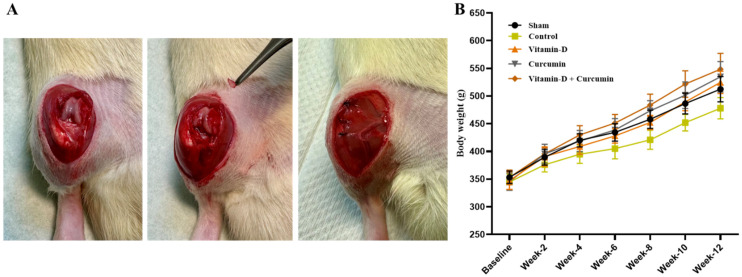
(**A**) Photograph of knee OA caused by ACLT + MMx surgery. (**B**) Body weight changes were determined for the sham group, control group, vitamin D group, curcumin group, and vitamin D + curcumin group (*n* = 6).

**Figure 3:**
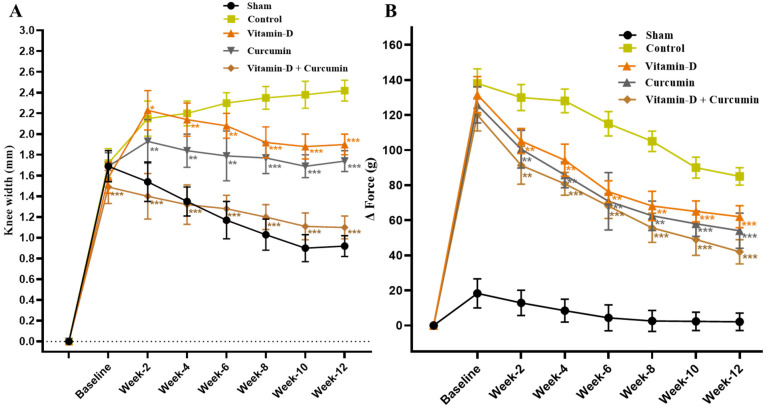
The effect of curcumin and vitamin D in ACLT + MMx-induced OA rats. (**A**) Knee width (mm) and (**B**) weight-bearing changes (*n* = 6). Data expressed as mean ± S.D. * *p* < 0.05, ** *p* < 0.01, and *** *p* < 0.001.

**Figure 4:**
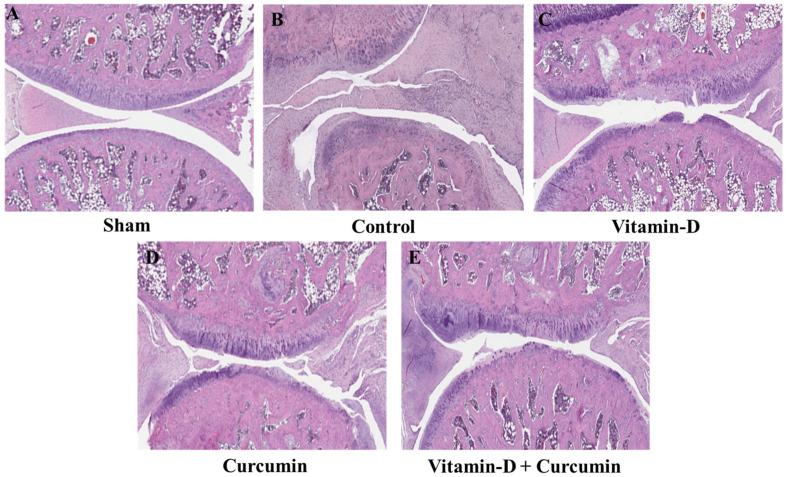
Histopathological changes in the OA knee joint. H and E assay of the cartilage protective effect of vitamin D and curcumin in ACLT + MMx-induced OA rats. (**A**) Sham group (*n* = 6), (**B**) control group (*n* = 6), (**C**) vitamin D group (*n* = 6), (**D**) curcumin group (*n* = 6), (**E**) vitamin D + curcumin group (*n* = 6), scale bar = 200 µM.

**Figure 5:**
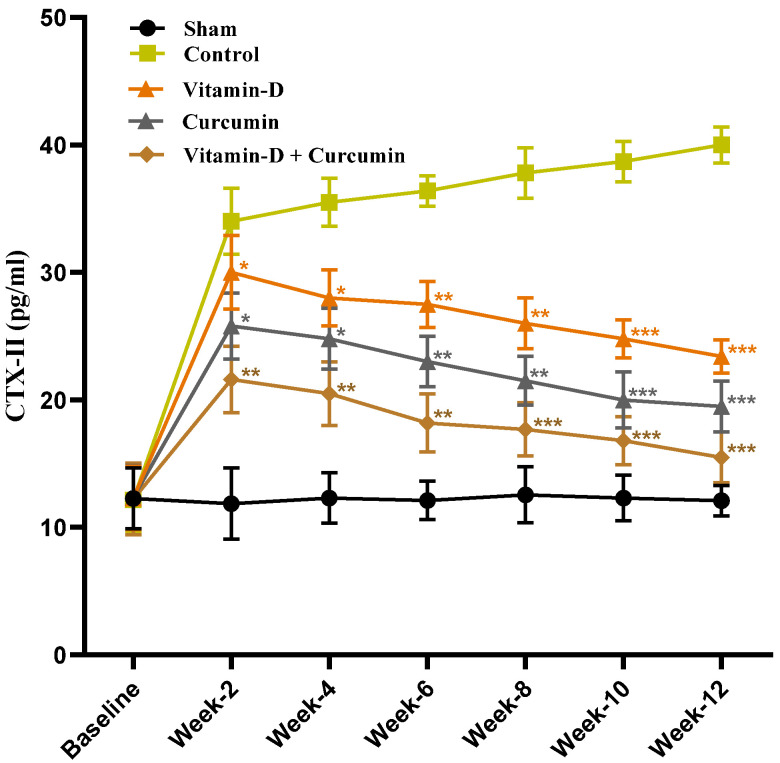
The serum ELISA studies of CTX-II (pg/mL) concentration in the sham, control, vitamin D, curcumin, and vitamin D + curcumin groups (*n* = 6). Data expressed as mean ± S.D. * *p* < 0.05, ** *p* < 0.01, and *** *p* < 0.001.

**Figure 6:**
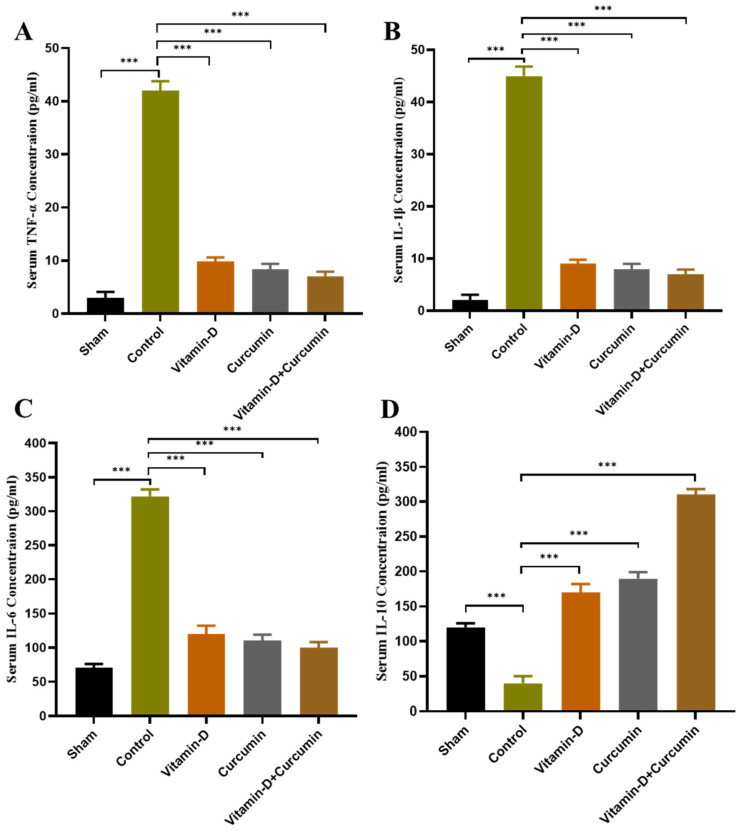
Vitamin D and curcumin on serum pro-inflammatory cytokines levels. ELISA studies of serum pro-inflammatory cytokines (**A**) TNF-α (pg/mL), (**B**) IL-1β (pg/mL), (**C**) IL-6 (pg/mL), and (**D**) IL-10 (pg/mL). Data expressed as means ± S.D (*n* = 6). *** *p* < 0.001.

**Figure 7:**
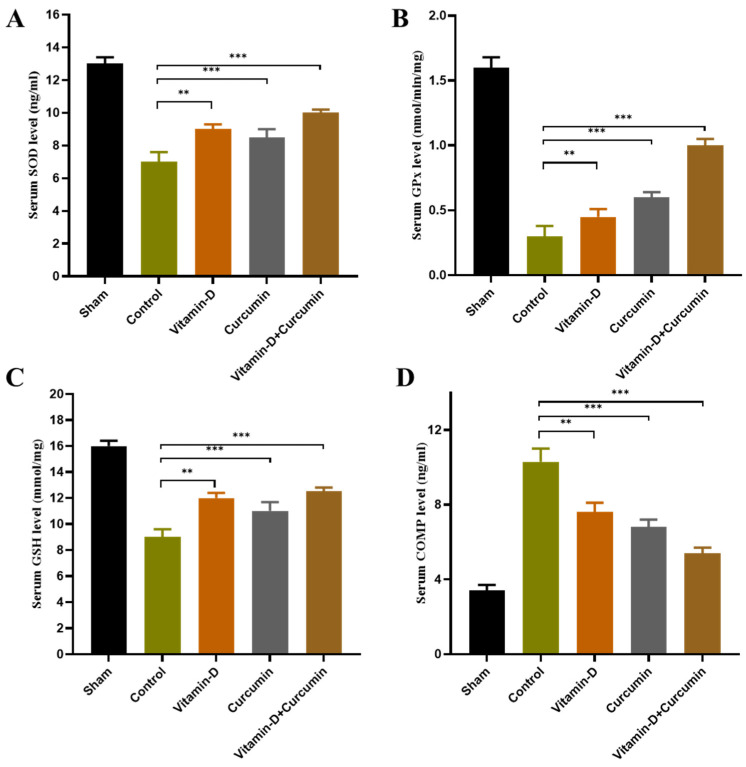
The antioxidative effect of vitamin D and curcumin was measured in all groups. The antioxidant biomarkers were measured as (**A**) SOD, (**B**) GPx, (**C**) GSH, and (**D**) COMP (*n* = 6). Data expressed as means ± S.D. ** *p* < 0.01 and *** *p* < 0.001.

**Figure 8:**
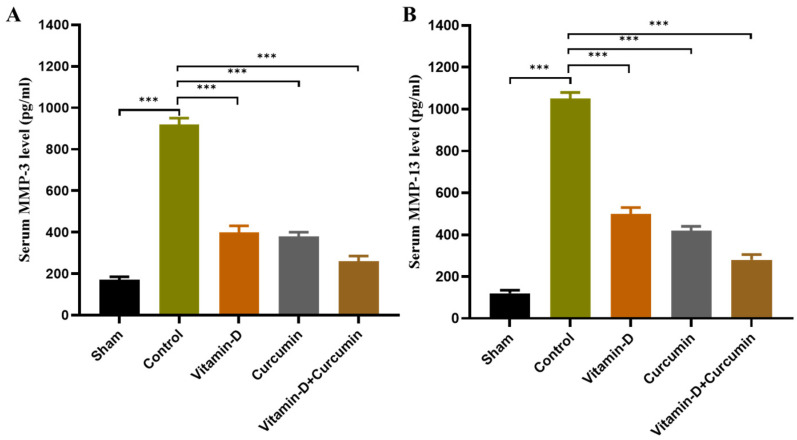
Vitamin D and curcumin on MMP levels in the serum were measured using ELISA analysis (*n* = 6). (**A**) MMP-3 (pg/mL) and (**B**) MMP-13 (pg/mL). Data expressed as means ± S.D. *** *p* < 0.001.

This study demonstrated that vitamin D and curcumin, particularly in combination, effectively reduced pain and inflammation in ACLT + MMx-induced knee OA in rats, providing enhanced cartilage protection and anti-inflammatory effects. Body weight increased consistently across groups without significant differences (Figure 2). Vitamin D, curcumin, and combined treatments reduced knee swelling and significantly improved weight bearing (Figure 3). Cartilage destruction and inflammation were minimized, with less degradation in vitamin D + curcumin-treated rats observed in H&E staining (Figure 4; Table 1). Serum CTX-II levels decreased, particularly in the combined treatment group, suggesting cartilage protection (Figure 5). The combined treatment improved anti-inflammatory and antioxidative responses by reducing pro-inflammatory cytokines TNF-α, IL-1β, and IL-6, and raising IL-10 (Figure 6), as well as enhancing antioxidant markers SOD, GSH, and GPx (Figure 7). MMP expression in chondrocytes was also reduced, supporting cartilage preservation (Figure 8). These findings suggest that vitamin D and curcumin, especially when combined, may offer a dual mechanism for knee OA management by promoting anti-inflammatory and antioxidative pathways.

### 3.1. Vitamin D and Curcumin Alleviate Pain in ACLT + MMx-Induced OA Rats

In the ACLT + MMx, on the right knee, induced knee OA in rats (Figure 2A). All groups of rats showed a constant increase in body weight with no statistical difference (Figure 2B).

The knee width (mm) in vitamin D-, curcumin-, and combined-treated rats was shown to be less swollen compared to water-treated OA control rats (Figure 3A). Moreover, when compared to sham group rats, vitamin D + curcumin-, curcumin-, and vitamin D-treated rats show a significantly reduced difference in weight bearing (∆ Force, in gm; Figure 3B).

### 3.2. Vitamin D and Curcumin Attenuate Cartilage Destruction in ACLT + MMx-Induced OA Rats

In OA knees, the destruction of articular cartilage is linked to more extracellular matrix degradation (ECM), less ECM production, and the loss of chondrocytes. Our previous study revealed that progressive reduction in serum vitamin D and increased MMPs contributed to the progression of knee OA [28]. The sham group shows normal healthy cartilage with intact structure and no significant signs of degradation. In contrast, the knee of control OA rats displays notable typical OA pathology with cartilage thinning, chondrocyte loss, and extracellular matrix disruption. Others demonstrate varying degrees of cartilage degradation, potentially reflecting the effects of vitamin D and curcumin treatments, which help reduce cartilage damage compared to untreated OA. For the chondroprotective property of vitamin D and curcumin, H&E staining revealed that vitamin D and curcumin treatment (Figure 4C–E) minimized articular cartilage destruction in comparison with the OA control group rats (Figure 4B). In contrast, the sham group had no destruction (Figure 4A). OARSI scores revealed that vitamin D- and curcumin-treated ACLT + MMx-induced rats (vitamin D, curcumin, and vitamin D + curcumin) had significantly less cartilage destruction than control group rats. H&E staining analysis revealed that the OA control group exhibited higher cartilage degradation and matrix loss scores compared to the sham group (Table 1).

### 3.3. Effect of Vitamin D and Curcumin on Serum CTX-II Levels in ACLT + MMx-Induced OA Rats

According to previous studies, CTX-II’s role in cartilage degradation is a critical marker for OA severity and the effect of treatment. Recent studies demonstrated an association of higher serum CTX-II with more severe progression of OA [6,53]. In our study, we found that serum CTX-II was lower in the vitamin D- and curcumin-treated groups in comparison with the OA control rats, and combined vitamin D and curcumin produced a better protective effect (Figure 5).

### 3.4. Effect of Vitamin D and Curcumin on Serum Cytokine Levels in ACLT + MMx-Induced OA Rats

Pro-inflammatory cytokines TNF-α, IL-6, IL-1β, and IL-10 promote articular cartilage matrix breakdown [14]. Recent studies supported this treatment strategy for OA by inhibiting the pro-inflammatory cytokine TNF-α, IL-1β, IL-6, and IL-10 serum levels [15]. In our study, vitamin D and curcumin remarkably inhibited the serum pro-inflammatory cytokines TNF-α, IL-1β, and IL-6 in comparison with the OA control group of rats (Figure 6A–C). In contrast, the anti-inflammatory cytokine (IL-10) expression showed an opposite expression to the pro-inflammatory cytokines, and the vitamin D and curcumin supplement increased the IL-10 serum level compared to that of OA control rats (Figure 6D). These findings indicated that the vitamin D and curcumin supplement inhibited inflammation in OA and reduced knee joint swelling.

### 3.5. Effect of Vitamin D and Curcumin on Antioxidant Expression in ACLT + MMx-Induced OA Rats

The pathogenesis of OA heavily relies on oxidative stress. Oxidative stress refers to the disparity between the formation of ROS and antioxidant defense mechanisms achieved through various molecules and enzymes such as SOD, GSH, and GPx. OA control animals had significantly lower serum GSH levels (Figure 7A–C) compared to sham group animals. Compared to the OA control group rats, vitamin D and curcumin treatment significantly increased GSH levels (*p* < 0.001). Similarly, the SOD level of OA control rats (Figure 7A) was lowered considerably compared to sham rats, and it was increased by the vitamin D + curcumin supplement compared to OA control rats (*p* < 0.001). In contrast, for COMP, the marker of cartilage turnover (Figure 7D), an increase in OA knee was observed, and vitamin D, curcumin, and both in combination reduced the COMP expression (*p* < 0.01).

### 3.6. Effect of Vitamin D and Curcumin on Serum MMPs in ACLT + MMx-Induced OA Rats

In chronic OA, pro-inflammatory cytokines often drive the production of MMPs, which degrade all components of the extracellular matrix (ECM). MMP-3 and MMP-13, key collagenases, play a pivotal role in collagen degradation and are critical in the progression of OA. MMP-13, produced by cartilage chondrocytes, contributes to ECM destruction by breaking down aggrecan and collagen, while MMP-3 targets non-collagen matrix components of joints [18,20,21]. Compared to the control group, rats treated with vitamin D, curcumin, or their combination showed significantly reduced levels of MMP-3 (Figure 8A) and MMP-13 (Figure 8B) enzymes. These results suggest that oral supplementation with vitamin D and curcumin effectively protects cartilage against collagen degradation by reducing MMP expression in joint chondrocytes.

## 4. Discussion

### 4.1. Anti-Inflammatory and Pain Relief Effects of Vitamin D and Curcumin

Although the results showed a consistent rise in body weight across all rat groups with no significant differences, weight gain might induce pain, as indicated by differences in the weight-bearing test between the two hindlimbs in control rats. This could also impact OA discomfort. The reduction in knee width in vitamin D + curcumin-treated rats compared to OA control rats indicated a significant decrease in knee inflammation, suggesting that combination therapy effectively suppressed inflammatory responses. This improvement in inflammation enhanced joint function and slowed OA progression. The reduced weight-bearing test (∆ Force, in gm) in the treatment groups compared to OA control rats demonstrated the therapeutic effects of vitamin D and curcumin in alleviating pain and joint swelling. The combination treatment was more effective than individual therapies, highlighting its superior anti-inflammatory and analgesic benefits. These findings underscore the potential of mixed natural compounds in managing OA symptoms and progression.

### 4.2. Protective Role of Vitamin D and Curcumin in Cartilage Preservation

The chondroprotective effect of vitamin D and curcumin in the ACLT + MMx-induced OA rat model was demonstrated by H&E staining. Vitamin D and curcumin supplementation significantly reduced articular cartilage destruction compared to the control group. The sham rats showed no cartilage destruction, with the greatest preservation observed in those receiving both treatments (Figure 4C–E). In contrast, OA control rats exhibited greater cartilage breakdown and matrix loss (Table 1). These findings highlight the potential of vitamin D and curcumin as therapeutic agents for knee OA, warranting further investigation of their anti-inflammatory and antioxidant properties for clinical application.

### 4.3. Vitamin D and Curcumin: Combating Inflammation and Cartilage Degradation

In this study, serum CTX-II levels, a key indicator of cartilage degradation, were significantly lower in the vitamin D, curcumin, and combination treatment groups compared to OA control rats (Figure 5). This demonstrates the efficacy of vitamin D and curcumin, individually and combined, in inhibiting cartilage breakdown. The reduction in CTX-II levels suggests that these compounds offer protective effects against cartilage degradation by mitigating inflammatory and catabolic processes. Furthermore, vitamin D, curcumin, and their combination significantly reduced pro-inflammatory cytokines TNF-α, IL-1β, and IL-6, while increasing anti-inflammatory cytokine IL-10 levels compared to OA control rats (Figure 6A–D). The decreased knee width in the treatment groups further supports their anti-inflammatory effects, indicating reduced joint swelling and overall inflammation. These findings highlight the potential of vitamin D and curcumin as therapeutic agents for managing OA by alleviating inflammation and preserving joint structure and function.

### 4.4. Reducing Oxidative Stress and Protecting Cartilage with Vitamin D and Curcumin

Our study showed that vitamin D and curcumin supplementation significantly inhibited oxidative stress in OA, as indicated by lower tissue levels of GSH in OA control rats compared to the sham group. Vitamin D and curcumin treatment significantly increased GSH expression (Figure 7C), suggesting improved antioxidant capacity. Additionally, GPx levels (Figure 7B), which were reduced in OA control rats, were significantly elevated following treatment. Similarly, SOD levels (Figure 7A), which were lower in OA control rats, increased significantly with vitamin D and curcumin supplementation. These findings demonstrate that the combined treatment restored antioxidant enzymes, reducing oxidative damage associated with OA. Furthermore, serum COMP levels (Figure 7D) were significantly reduced in the vitamin D + curcumin group, supporting the therapeutic potential of this combination. Overall, vitamin D and curcumin enhanced oxidative stress defense mechanisms and modified cartilage biomarkers, highlighting their role in OA management. The treatment also effectively reduced MMP-13 and MMP-3 levels (Figure 8A,B), key enzymes involved in cartilage degradation. By inhibiting these catabolic enzymes in joint chondrocytes, vitamin D + curcumin mitigated cartilage damage and preserved joint integrity. These results underscore the potential of vitamin D + curcumin as a therapeutic strategy to protect cartilage and counteract OA progression by regulating key matrix-degrading enzymes.

This study highlights the potential of combining vitamin D and curcumin to manage and prevent knee OA by reducing inflammatory markers such as TNF-α, IL-1β, and IL-6 and combating oxidative stress. These compounds preserve cartilage, improve joint function, and slow OA progression, with the combination showing synergistic effects surpassing individual treatments and delaying the need for invasive procedures like joint replacement. Despite these promising findings, limitations include the use of a rat model, differences from human physiology and lifestyle factors, and controlled experimental conditions that may limit real-world applicability. This study provides evidence that vitamin D and curcumin effectively reduce inflammatory markers and enhance antioxidant defenses, offering a non-invasive approach to improve joint health and systemic inflammation. Furthermore, the combination demonstrated superior anti-inflammatory effects compared to prior studies, significantly reducing pro-inflammatory markers and increasing IL-10 levels, suggesting a more effective strategy for managing inflammation and delaying OA progression.

## 5. Conclusions

Our results demonstrate that vitamin D and curcumin supplement effectively reduced knee OA progression and reduced pain, making them promising options for early OA treatment. In our study, both vitamin D and curcumin, especially when used together, protect cartilage in OA rats by reducing inflammation and cartilage degradation. In ACLT + MMx-induced OA rats, low serum levels of vitamin D and curcumin, along with high CTX-II, indicated cartilage damage. After an oral supplement of vitamin D and curcumin, serum vitamin D and curcumin levels were raised and CTX-II levels were reduced in OA rats, suggesting cartilage protection. In H&E studies, vitamin D + curcumin treatment significantly reduced cartilage destruction. ELISA tests also showed that serum levels of the pro-inflammatory cytokines TNF-α, IL-1β, and IL-6 dropped substantially after treatment with vitamin D + curcumin. For anti-inflammatory cytokines, the vitamin D and curcumin supplement increased IL-10 serum levels compared to OA control rats. Vitamin D and curcumin may help manage knee OA by reducing inflammation and oxidative stress, thus improving joint health and pain reduction. In addition, vitamin D + curcumin stopped the expression of MMPs in chondrocytes (MMP-3 and MMP-13), which are needed to protect cartilage from the harsh conditions of knee OA. This study shows that vitamin D + curcumin has cartilage protection and pain reduction effects and can be a supplement for clinical knee OA management.

## Figures and Tables

**Table 1:** Histology OARSI scoring of OA knee joint. Data expressed as mean ± S.D. * *p* < 0.05, ** *p* < 0.01, and *** *p* < 0.001.

	Sham (*n* = 6)	Control (*n* = 6)	Vitamin-D (*n* = 6)	Curcumin (*n* = 6)	Vitamin-D + Curcumin (*n* = 6)
1. Cartilage matrix loss (mm)	0 **	4.0 ± 0.6	3.2 ± 0.6	1.6 ± 0.3	1.2 ± 0.2
2. Cartilage degeneration score	0 ***	10.4 ± 1.2	6.18 ± 2.4 *	3.2 ± 1.1 **	3.3 ± 0.6 **
3. Total cartilage degeneration width (mm)	0 ***	2.31 ± 0.4	1.87 ± 0.4	1.81 ± 0.2	1.14 ± 0.2 *
4. Significant cartilage degeneration width (mm)	0 ***	1.8 ± 0.21	0.72 ± 0.3 *	0.18 ± 0.1 ***	0.23 ± 0.1 ***
5. Zonal depth ratio of lesions	0 **	2.2 ± 0.4	0.7 ± 0.4 *	0.52 ± 0.3 **	0.46 ± 0.2 **

## Data Availability

Data will be made available upon request. The data have not been made publicly available due to privacy and ethical considerations.
